# Evaluation of an Online System for Routine Outcome Monitoring: Cross-sectional Survey Study

**DOI:** 10.2196/29243

**Published:** 2021-12-01

**Authors:** Deanna E Wiebe, Shannon Remers, Pria Nippak, Julien Meyer

**Affiliations:** 1 Department of Health Services Management Ryerson University Toronto, ON Canada; 2 Homewood Health Inc Guelph, ON Canada

**Keywords:** routine outcome monitoring, progress monitoring and feedback, outcome measures, web-based outcome monitoring, routine outcome monitoring software, outcome measurement questionnaire, measurement-based care

## Abstract

**Background:**

The use of routine outcome monitoring (ROM) in the treatment of mental health has emerged as a method of improving psychotherapy treatment outcomes. Despite this, very few clinicians regularly use ROM in clinical practice. Online ROM has been suggested as a solution to increase adoption.

**Objective:**

The aim of this study is to identify the influence of moving ROM online on client completion rates of self-reported outcome measures and to identify implementation and utilization barriers to online ROM by assessing clinicians’ views on their experience using the online system over previous paper-based methods.

**Methods:**

Client completion rates of self-reported outcome measures were compared pre- and postimplementation of an online system of ROM. In addition, a survey questionnaire was administered to 324 mental health service providers regarding their perception of the benefits with an online system of ROM.

**Results:**

Client completion rates of self-reported measures increased from 15.62% (427/2734) to 53.98% (1267/2347) after they were moved online. Furthermore, 57% (56/98) of service providers found the new system less time-consuming than the previous paper-based ROM, and 64% (63/98) found that it helped monitor clients. However, the perceived value of the system remains in doubt as only 23% (23/98) found it helped them identify clients at risk for treatment failure, and only 18% (18/98) found it strengthened the therapeutic alliance.

**Conclusions:**

Although the current study suggests mixed results regarding service providers’ views on their experience using an online system for ROM, it has identified barriers and challenges that are actionable for improvement.

## Introduction

The prevalence of mental illness, accompanied by its social and economic burden on the individual and society, has gained global recognition [1,2], creating a push to invest in solutions [3]. Mental health action plans that include evidence-based interventions with measurable outcomes have been identified as important components of future improvements to mental health services [3]. The incorporation of routine outcome monitoring (ROM) into clinical practice has emerged as a method of improving psychotherapy treatment outcomes [4]. ROM involves monitoring client progress throughout their course of treatment at regular intervals using standardized measures and feeding the information back in real time to the clinician and client, thereby allowing for the identification of any need to change the care plan [4-6]. According to Lambert and Harmon [7], client progress measurements and feedback appear to work similarly to a physician monitoring patients’ blood sugar in managing their diabetes, and most importantly, it can identify possible impending treatment failure. Studies have shown that ROM contributes to an increased number of clients who improve from the receipt of mental health treatment [8], an increase in the degree to which they improve, and a decrease in the number of treatment failures [7,9].

The use of ROM in clinical settings appears to have a positive effect on patient outcomes in several ways. One key benefit is that ROM can help clinicians identify clients at risk for treatment failure by limiting the effect of overestimating their own abilities (self-assessment bias) [10-12]. Boswell et al [13] state, “Clinicians could benefit from using tracking systems because of their likely overly optimistic estimates of their clients’ outcome and their inability to predict treatment failure, specifically, reliable negative change.” A study by Hannan et al [14] which examined the ability of therapists (clinicians) to identify patient deterioration found that therapists only identified 2.5% (1 of 40) of clients who left treatment worse than when they began. Similarly, Lambert [10] states, “…a significant therapy-related cause of poor outcomes is the failure of therapists to be aware of poor treatment response as it develops over the course of therapy.” When clinicians can identify clients at risk for treatment failure earlier, they can adjust the course of treatment or optimize treatment instead of waiting until the end of treatment [15].

Another positive effect of ROM in mental health treatment is quicker client improvement, which is tied to faster recovery resulting in fewer treatment sessions [14,15]. Fewer treatment sessions mean cost savings for health institutions facing increasing pressures for accountability and cost containment [16]. A quick recovery also means less suffering on the part of the client [16]. ROM with ongoing client feedback is therefore a method of providing more efficient and cost-effective care [15,17].

The use of ROM can also improve the therapeutic alliance between clinician and client. Clinicians who form stronger alliances with their clients can expect better outcomes [14,15,18-21]. Brattland et al [22] found that when ROM was in place, alliance ratings increased more than they did with the treatment-as-usual condition, and this improvement in the therapeutic alliance resulted in less posttreatment impairment.

Despite these benefits of ROM and the fact that many countries are recommending the use of ROM in various mental health settings [10,23-25], previous studies assessing usage suggest that fewer than 14% of clinicians use standardized progress monitoring measures regularly in their provision of mental health services [22,26]. Several obstacles faced by mental health organizations and therapists in implementing ROM in clinical practice may explain the low rates of usage: the time required to administer, score, interpret and report client feedback; the financial burden of implementation; multiple stakeholders with different needs; and philosophical barriers, such as scepticism regarding the relevancy and utility of the measurement tools, fear and mistrust about what the data will be used for, fear of being monitored, and privacy and ethical concerns [12,27,28]. Inadequate training and awareness regarding the use of ROM, how to complete the measures, and a lack of ongoing technical support, compound these issues [27,29].

Previous studies evaluating the organizational benefits of ROM have shown that using online systems provides instantaneous feedback to clinicians and clients [5,7,8,12]. The Partners for Change Outcome Measurement System, which uses web-based software to calculate and track the client’s outcome rating score or the OQ-Analyst software signal alert system, has demonstrated a reduction in client deterioration rates and significant change in clients predicted to have a poor outcome [6]. Barriers to uptake of the use of ROM can also be overcome by implementing a system that is simple and easy to use, is not disruptive to routine mental health therapy practice, and that can “expedite and ease practical difficulties” [12].

The objective of this paper is to identify if moving to an online system of ROM influences client completion rates of the outcome questionnaires. In addition, service providers’ views on their experience using an online system will be examined to identify implementation and utilization barriers and highlight actionable items where improvements can be made to ensure successful future implementations.

## Methods

The study was conducted at a large mental health and addiction facility in Canada, and 2 types of data were collected. First, retrospective data were gathered on completion of self-reported measures by clients. Second, a cross-sectional survey of mental health service providers was conducted to investigate their perception of the benefits of using an online ROM in their treatment of clients in the Depression Care and Trauma Care individual outpatient counseling programs. For the purposes of this study, clinicians are referred to as “service providers.” A research ethics board application was submitted, and ethics review was deemed to not be required.

In this study, ROM refers to the repeated measurement of a client’s progress over the course of treatment according to standardized self-reported measurement questionnaires or assessments (see Multimedia Appendix 1 for a list of assessments and their expected frequency). This information regarding the client’s current status is fed back to the clinician or client and is intended to be used by the clinician to assess if a change or alteration in the current treatment plan is necessary [5,7,30]. The self-reported questionnaires commonly measure client progress regarding symptom severity, social functioning, and personal well-being [9,14,28,31].

Before the online ROM was implemented, the self-reported measures (Multimedia Appendix 1), were completed by the service providers in a session with the client and were manually entered into the client system by the service provider or faxed to the main office administration staff for manual entry into the client system. Postimplementation of the online system for ROM, the same assessments were performed at the same frequency and intervals (Multimedia Appendix 1), but they were to be completed by the client prior to coming to session via their online portal. In this way, feedback would be available in real time to both the service provider and the client.

Rates of completion of the self-reported measurement questionnaires (assessments) by the clients as part of the ROM process were calculated as follows: each client that filled out at least 1 assessment battery at 1 time point during the course of treatment was calculated as 1 client with a completed assessment, both pre- and postimplementation. The total number of clients with a completed assessment was divided by the total number of clients. All clients in the Depression Care and Trauma Care programs were expected to complete assessments; all were included for the time period of 18 months prior to the implementation of the online ROM and again at 18 months postimplementation of the online ROM.

A survey questionnaire was designed by the investigators for this study based on a literature review of research on utilization and attitudes toward ROM in the treatment of mental health and adaptation of the survey questions used in those studies [26,30,32,33].

The questionnaire consisted of ten, 5-point Likert scale questions, asking service providers how they would best characterize their feelings toward the use of an online ROM system in the areas of time savings (Q1), client receptiveness (Q2), allowance for regular progress monitoring of clients (Q3), adequacy of training (Q4), strengthening of the therapeutic alliance (Q5), identification of clients at risk for failure (Q6), increased workload (Q7), help available as needed to assist in using the program (Q8), if confidence in usage increased over time (Q9), and if it positively impacted the care they provided (Q10). In addition, the questionnaire included 1 open-ended free-text question asking for further comments. The questionnaire was offered in both English and French to accommodate the bilingual nature of health care services in Canada (see the English version of the service provider survey in Multimedia Appendix 2). The data collection for the survey occurred between January 20, 2020, and February 20, 2020. Two reminder emails to complete the survey were sent, the first halfway through the data collection period on February 3, 2020, and the second 2 weeks after that on February 17, 2020.

The online survey questionnaire created and used for this study was distributed to 324 mental health service providers via email. All service providers were mental health counselors and had at least a master’s degree in social work, psychology, or another health-related discipline. The inclusion criteria for service providers were that they had an online account and that they had at least 1 client using the program at the time of study. Of the 324 service providers who received the survey, 98 completed the questionnaire, resulting in a response rate of 30.2% (98/324). Of the 98 responses, 10 were completed in French.

## Results

Client completion rates (total number of clients with at least 1 completed assessment divided by the total number of clients) of the self-reported measurement questionnaires using paper-based methods calculated over an 18-month period preimplementation of an online ROM were 15.62% (427/2734). Postimplementation of an online ROM, client completion rates calculated over an 18-month period were 53.98% (1267/2347).

Survey responses provided the following results. Most service providers (56/98, 58%), responded that the online system was less time-consuming than were previous paper-based methods (Q1); however, only 31 out of 98 (31%) agreed that the system did not increase their workload (Q7).

With regard to the online system of ROM allowing for regular progress monitoring of their clients (Q3), 63 out of 98 (64%) service providers responded that the online system did allow for regular progress monitoring of their clients. However, only 23 out of 98 (23%) responded that the online system helped identify earlier clients at risk for treatment failure (Q6), and only 18 out of 98 (18%) responded that the online system strengthened the therapeutic alliance with their clients (Q5). In addition, only 45 out of 98 (64%) service providers responded that their clients were receptive to using the online system to complete the self-assessment questionnaires (Q2), and only 38 out of 98 (38%) responded that the program positively impacted the care they provided (Q10).

**Figure 1:**
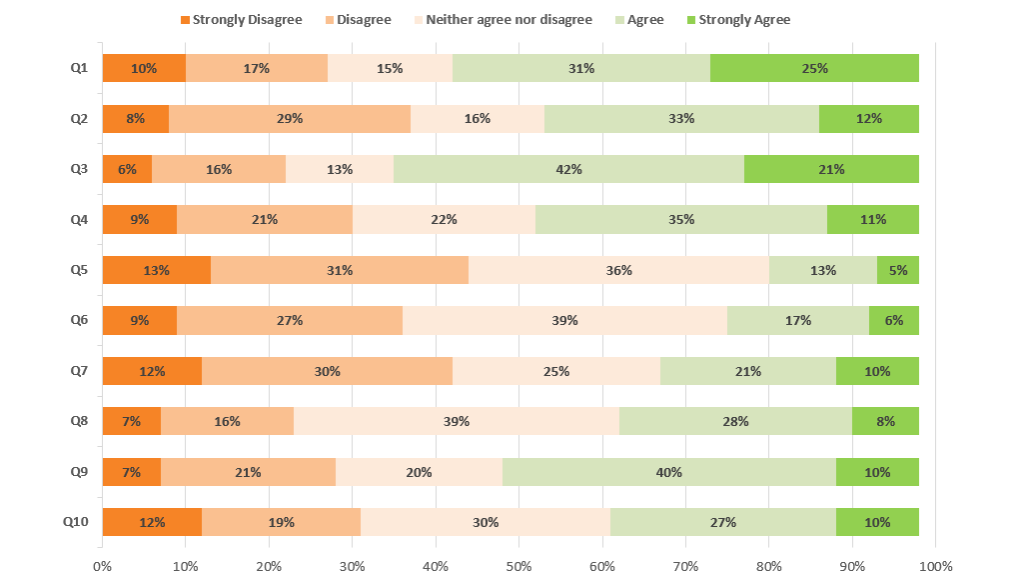
Service Provider Survey Results.

With regard to their ability to successfully use the system, only 36 out of 98 (37%) reported that help was available as needed to assist with using the program (Q8), 46 out of 98 (47%) responded that they received adequate training (Q4), and 50 out of 98 (51%) felt more confident in their ability to use the system since it was first introduced (Q9; Figure 1).

Many of the 48 free-form responses were able to be split into several themes. The service providers who responded with positive feedback indicated their appreciation for the instant feedback on the progress of their clients: “I would say the best part is that it’s easier than paper and results are easy/instant”; *“*I like the paper savings, instant scoring and ability to visually monitor progress online*”*; and *“*Overall I think it is a great tool…*”.*

Most service providers, however, stated difficulties navigating the software and understanding the process. Many of these respondents specifically stated that they received inadequate training, and many made a request for additional training. Due to their own difficulties using the software, some also described issues with assisting their clients in successfully using the software. In addition, some service providers stated they had to resort back to completing the measures for their clients or completing it with their clients on paper in session and faxing in the results. Reasons given by service providers for this were clients reporting technical difficulties, preferring paper and pen, reporting being too depressed already, or stating the process was frustrating and anxiety-provoking. Some service providers stated they found no benefit with using the online program over previous paper-based methods, some reported an increase in their workload, and some reported accessibility issues due to the platform not being entirely in French.

## Discussion

### Principal Findings

The findings of this study suggest that an online system supports adoption of routine outcome monitoring in the treatment of mental health. Despite this study’s findings of low client receptiveness with service providers occasionally having to revert to paper-based completion of the measures, client completion rates increased significantly with the use of the online system of ROM. Previously with a solely paper-based system, only 16% of clients completed assessments; however, after implementation of the online system, the completion rates increased to 54%. A study evaluating the benefits, barriers, and disadvantages of electronic patient-reported outcome measures identified that electronic collection offers more advantages over paper-based methods. The study, conducted via a systematic review of articles that evaluated electronic patient-reported outcome measures identified advantages that included greater patient preference, lower costs, faster completion time, higher data quality, and higher response rates [34].

Upon examination, the benefits of an online system over paper-based methods are clear; however, service providers’ perceptions are mixed. Contradictory results were found with regards to efficiency. Although 56 out of 98 (57%) service providers responded that the online system was less time-consuming than were previous paper-based methods, only 31 out of 98 (31%) responded that the system did not increase their workload. One of the main barriers identified in previous literature to the utilization of ROM has been the significant time burden involved, and it has been suggested that software systems may alleviate some of the burden [5,12,35,36]. The results of the current study, with 58% (56/98) of service providers responding that the online system was less time-consuming, show promise for improvement in ROM utilization if an online system is adopted. However, the low response rate of only 31% (31/98) of service providers reporting that the online system did not increase their workload is concerning.

With regard to the service providers’ views on their experience using an online system of ROM, 63 out of 98 (64%) agreed that the system of the online ROM did allow for regular progress monitoring of their clients. However, only a minimal number of service providers recognized benefits with the use of an online ROM for identifying clients at risk for treatment failure (23/98, 23%) or strengthening the therapeutic alliance (18/98, 18%). The online system of ROM that was implemented allowed service providers and clients to view progress in graphs in real time. The service provider was then able to determine if the client’s progress was going in a positive or negative direction. The online system also had a dashboard flag to allow the service provider to identify if the client was suicidal or displaying suicidal ideation. This flag is similar to other ROM systems such as the OQ-Analyst feedback system that provides a red alert signal to indicate that the client is responding poorly to treatment [6]. It may be that service providers are relying on their own abilities and efforts to identify clients at risk for treatment failure or to strengthen the therapeutic alliance rather than using the tools available to them with the online system. This has been demonstrated in previous literature which reports that clinicians have an overly positive self-assessment bias as to their ability to affect client improvement and a tendency to underestimate client deterioration [11,37]. Increase in training and future evaluation to assess if service providers are viewing the real-time feedback that is available to them through the online system should be completed to improve these 2 study results.

Only 45 out of 98 (46%) service providers reported that their clients were receptive to using the online system. This finding was underscored in the open-ended comments with some indicating that their clients refused to use the program, preferred paper and pen, or preferred to complete the questionnaires in session with the therapist. Clients also reported they were too depressed or lacked the motivation to use the program or felt the program provoked feelings of frustration, agitation, and anxiety. Similar findings regarding client receptiveness have been identified in the literature with some users expressing frustrations with a complicated or unintuitive interface, or technical issues and malfunctioning websites to the point of giving up [38]. In a 2021 research study performed in Australia to improve mental health and well-being health information technology for culturally diverse youth in nonurban areas, participants identified that the technology should be easy to use and understand and should not make the user feel overwhelmed or frustrated [39]. Functionality has been demonstrated in the literature to be of high importance in enhancing user satisfaction for the implementation of a web-based platform [40]. Overall, our findings are consistent with previous research findings and suggest a lack of user-friendliness may be a factor in the low client receptiveness to using the ROM online system in our current study.

Finally, our study showed low results regarding service providers receiving adequate training or having help available when using the system was needed. Previous research concurs with these results in that it has been identified that lack of proper training and support is a barrier to successful implementation and utilization of a ROM program and that both these components are needed for successful adoption of an online system [27]. Therefore, enhancement in training and ongoing technical support could be actioned to improve success of future implementations.

### Limitations

A limitation in this study is the low survey response rate by service providers, with just 98 out of 324 (30.2%) participants responding. Although this response rate and sample size are comparable to those of previous survey studies [30,41], obtaining feedback from more service providers using the online system would give a truer picture of the perception of benefits. Another limitation relates to the client completion rates, as the clients only had to fill out 1 assessment battery at 1 time point during the course of treatment to be included in the calculation, which does not necessarily give a true picture of increased rates. Further studies should investigate whether online ROM leads to a sustained completion of assessments.

For the purpose of parsimony, each question investigated a different construct. Future studies should use multiple items to enable a measurement of reliability and validity. Recommendations for future research also include an expansion of sociodemographic characteristics of service providers, which may help to explain the current findings. For example, service provider age, ethnicity, length of employment, years of experience, education details, and physical location they are servicing could be included as factors that may influence perceptions of the use of an online system for ROM. Previous research suggests that mental health therapists who have graduated more recently tend to value ROM and are more inclined to use it [25].

A direct survey evaluation of client experience and perceptions using an online system for completing mental health questionnaires is also recommended for future research given the current low receptiveness of clients. This additional information would be beneficial in highlighting necessary improvements that should be made for future implementations. It also would be helpful to reevaluate service providers’ perceptions on the use of an online ROM after an enhancement is made in training and ongoing support to identify if this adjustment would glean improved results.

### Conclusions

Overall, the findings of this study suggest that online ROM has strong potential to lead to increased adoption of ROM, which has been associated with better outcomes for patients. This should encourage researchers and practitioners to identify and address the barriers and challenges, which, with limited intervention, could increase the chances of future implementation success, improved utilization, and completion of the measures.

## Appendix

Multimedia Appendix 1 Routine outcome monitoring (ROM) self-reported measurement questionnaires.

Multimedia Appendix 2 Service provider survey.

## Abbreviations

ROM: routine outcome monitoring
