# Supplementary material for: Evaluation of an Online System for Routine Outcome Monitoring: Cross-sectional Survey Study
Source: JMIR Ment Health. 2021 Dec 1;8(12):e29243. doi: 10.2196/29243 (PMC8686458; doi:10.2196/29243)
Supplement: Multimedia Appendix 1 [file mental_v8i12e29243_app1.docx]

**Multimedia Appendix 1**

**ROM Self-Reported Measurement Questionnaires (Assessments) used for Depression Care (DC) and Trauma Care (TC)**

| **Assessment** | **Frequency** |
| --- | --- |
| DASS-21 (Depression Anxiety Stress Scale) | Baseline and every 4 weeks |
| WOS (Workplace Outcome Suite) | Baseline and every 4 weeks |
| PCL-5 (PTSD Checklist for DSM-V) | Baseline and every 4 weeks (only for trauma care patients) |
