# Supplementary material for: Evaluation of an Online System for Routine Outcome Monitoring: Cross-sectional Survey Study
Source: JMIR Ment Health. 2021 Dec 1;8(12):e29243. doi: 10.2196/29243 (PMC8686458; doi:10.2196/29243)
Supplement: Multimedia Appendix 2 [file mental_v8i12e29243_app2.docx]

**Multimedia Appendix 2**

**Service Provider Survey**

For each of the questions below, circle the response that best characterizes how you feel about the statement, where 1 = Strongly Disagree, 2 = Disagree, 3 = Neither Agree nor Disagree, 4 = Agree, 5 = Strongly Agree.

|  |  | **Strongly Disagree** | **Disagree** | **Neither Agree nor Disagree** | **Agree** | **Strongly Agree** |
| --- | --- | --- | --- | --- | --- | --- |
| Q1 | The online system is less time consuming than paper-based questionnaires | 1 | 2 | 3 | 4 | 5 |
| Q2 | Clients were receptive to using the online system | 1 | 2 | 3 | 4 | 5 |
| Q3 | The online system allowed for regular progress monitoring of my clients | 1 | 2 | 3 | 4 | 5 |
| Q4 | I received adequate training on how to use the online system | 1 | 2 | 3 | 4 | 5 |
| Q5 | The online system strengthened therapeutic alliance with my clients | 1 | 2 | 3 | 4 | 5 |
| Q6 | The online system helped identify earlier the clients at risk for treatment failure | 1 | 2 | 3 | 4 | 5 |
| Q7 | The online system did not increase my workload | 1 | 2 | 3 | 4 | 5 |
| Q8 | Help was available as needed to assist in using the online system | 1 | 2 | 3 | 4 | 5 |
| Q9 | I feel more confident in my ability to use the online system since it was first introduced | 1 | 2 | 3 | 4 | 5 |
| Q10 | Overall, the addition of the online system has positively impacted the care I provide | 1 | 2 | 3 | 4 | 5 |

Do you have any further comments about the online system? Please type your answer in the space provided:
